# 4-Nitro-1-[(trimethyl­sil­yl)ethyn­yl]benzene: low-temperature polymorph at 100 K^1^

**DOI:** 10.1107/S1600536812034034

**Published:** 2012-08-08

**Authors:** Jasmine N. Millican, Frank R. Fronczek, Steve F. Watkins

**Affiliations:** a Department of Chemistry, Louisiana State University, Baton Rouge, LA 70803-1804, USA

## Abstract

The title compound, C_11_H_13_NO_2_Si, is a low-temperature form of the previously reported room-temperature structure [Garcia *et al.* (1998 ▶). *Acta Cryst.* C**54**, 489–491]. At 298 K, the material crystallizes in the space group *Pnma* and occupies a crystallographic mirror plane, but at 100 K the space group changes to *P*2_1_2_1_2_1_, the volume decreases by 5% and the mol­ecule distorts. The greatest mol­ecular distortions from *C*_s_ symmetry are rotations of the trimethyl­silyl and nitro groups by 10.56 (8) and 11.47 (9)°, respectively, to the benzene mean plane. At low temperature, the crystal also becomes an inversion twin, the refined ratio of the twin components being 0.35 (15):0.65 (15).

## Related literature

For the synthesis of the title compound, see: Takahashi *et al.* (1980 ▶). For the crystal structure of the room temperature form of the title compound, see: Garcia *et al.* (1998 ▶). For a description of the Cambridge Structural Database, see: Allen (2002 ▶). For Hooft analysis of Bijvoet pairs, see: Hooft *et al.* (2008 ▶).
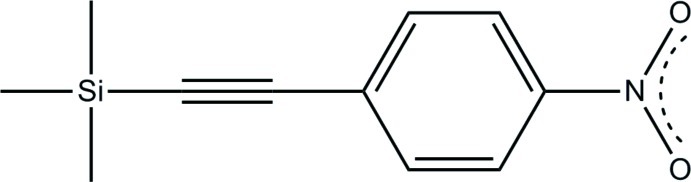


## Experimental

### 

#### Crystal data

C_11_H_13_NO_2_Si
*M* *_r_* = 219.31
Orthorhombic, 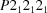

*a* = 10.222 (2) Å
*b* = 7.128 (2) Å
*c* = 16.537 (4) Å
*V* = 1204.9 (5) Å^3^
*Z* = 4
Mo *K*α radiation
μ = 0.18 mm^−1^
*T* = 100 K
0.20 × 0.15 × 0.12 mm

#### Data collection

Nonius KappaCCD diffractometer
Absorption correction: multi-scan (*SCALEPACK*; Otwinowski & Minor, 1997 ▶) *T*_min_ = 0.966, *T*_max_ = 0.979
17805 measured reflections
4690 independent reflections
3538 reflections with *I* > 2σ(*I*)
*R*_int_ = 0.032

#### Refinement

*R*[*F* ^2^ > 2σ(*F* ^2^)] = 0.051
*wR*(*F* ^2^) = 0.123
*S* = 1.02
4690 reflections
139 parameters
H-atom parameters constrained
Δρ_max_ = 0.36 e Å^−3^
Δρ_min_ = −0.32 e Å^−3^
Absolute structure: Flack (1983 ▶), 1953 Bijvoet pairs
Flack parameter: 0.35 (15)

### 

Data collection: *COLLECT* (Nonius, 2000 ▶); cell refinement: *DENZO* and *SCALEPACK* (Otwinowski & Minor, 1997 ▶); data reduction: *DENZO* and *SCALEPACK*; program(s) used to solve structure: *SIR2002* (Burla *et al.*, 2003 ▶); program(s) used to refine structure: *SHELXL97* (Sheldrick, 2008 ▶); molecular graphics: *ORTEP-3 for Windows* (Farrugia, 1997 ▶); software used to prepare material for publication: *WinGX* (Farrugia, 1999 ▶).

## Supplementary Material

Crystal structure: contains datablock(s) global, I. DOI: 10.1107/S1600536812034034/su2491sup1.cif

Structure factors: contains datablock(s) I. DOI: 10.1107/S1600536812034034/su2491Isup2.hkl

Supplementary material file. DOI: 10.1107/S1600536812034034/su2491Isup3.cml

Additional supplementary materials:  crystallographic information; 3D view; checkCIF report

## Figures and Tables

**Fig. 1.**
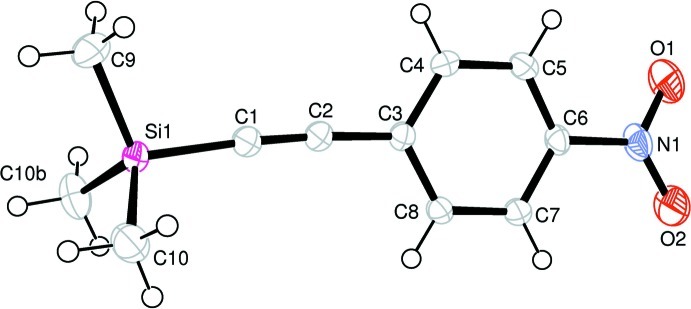
View of the molecular structure of the title compound, with atom numbering. The displacement ellipsoids are drawn at the 50% probability level.

## supplementary crystallographic information

### Comment

At 298 K title molecule has C_s_ symmetry (Garcia *et al.*, 1998; CSD refcode NILWIO; Allen, 2002), with all but two methyl groups lying in the mirror plane. When the temperature is reduced to 100 K, the inversion center vanishes and the space group changes from *Pnma* to *P*2_1_2_1_2_1_, the unit cell volume is reduced from 1261.8 (3) to 1204.9 (5) Å^3^, and the molecular symmetry is reduced to C_1_. The mean plane of the six benzene C atoms, (mean deviation δ_r.m.s._ = 0.004 Å), is normal to the *b* axis at 298 K but inclines +4.51 (8)° from normal at 100 K, while the C1—Si1—C9 plane is rotated off-normal by -6.05 (8)° and the NO_2_ group is rotated off-normal by +15.78 (9)°.

Other geometrical parameters in the two polymorphs are similar, for example [those at 298 K are in parentheses] : C1≡C2 = 1.209 (2) [1.199 (4)] Å, Si1—C1 = 1.845 (2) [1.839 (3)] Å, Si1—C9 = 1.856 (2) [1.831 (4)] Å, Si1—C10 = 1.853 (2) [1.838 (3)] Å, Si1—C10B = 1.856 (2)[1.838 (3)] Å, N1—O1 = 1.219 (2) [1.201 (4)] Å, N1—O2 = 1.221 (2) [1.175 (4)] Å, Si1—C1≡C2 = 176.1 (2) [177.9 (3)]°, C1≡C2—C3 = 175.9 (2) [178.0 (3)]°, O1—N1—O2 = 123.5 (2) [122.9 (3)]°.

### Experimental

The compound was prepared by palladium(II) coupling of trimethylsilylacetylene with 4-nitroiodobenzene as described by (Takahashi *et al.*, 1980).

### Refinement

Analysis of 1953 Bijvoet pairs yields a Hooft (Hooft *et al.*, 2008) parameter *y* = 0.42 (6) and P3(rac-twin) = 1.000. The model was therefore refined as an inversion twin (*SHELXL* commands TWIN and BASF) giving the refined ratio 0.35 (15):0.65 (15) for the twin components. The C-bound H atoms were placed in calculated positions, guided by difference maps, and treated as riding atoms: C—H = 0.95 and 0.98 Å for CH(aromatic) and CH_3_ H atoms, respectively, with *U*_iso_ = k × *U*_eq_(C), where k = 1.5 for CH_3_ H atoms and = 1.2 for other H atoms. A torsional parameter was refined for each methyl group.

### Crystal data

**Table:**

C_11_H_13_NO_2_Si	*F*(000) = 464
*M**_r_* = 219.31	*D*_x_ = 1.209 Mg m^−^^3^
Orthorhombic, *P*2_1_2_1_2_1_	Mo *K*α radiation, λ = 0.71073 Å
Hall symbol: P 2ac 2ab	Cell parameters from 2709 reflections
*a* = 10.222 (2) Å	θ = 2.5–34.3°
*b* = 7.128 (2) Å	µ = 0.18 mm^−^^1^
*c* = 16.537 (4) Å	*T* = 100 K
*V* = 1204.9 (5) Å^3^	Needle fragment, yellow
*Z* = 4	0.20 × 0.15 × 0.12 mm

### Data collection

**Table:**

Nonius KappaCCD diffractometer	4690 independent reflections
Radiation source: sealed tube	3538 reflections with *I* > 2σ(*I*)
Horizonally mounted graphite crystal monochromator	*R*_int_ = 0.032
Detector resolution: 9 pixels mm^-1^	θ_max_ = 33.8°, θ_min_ = 3.1°
ω and φ scans	*h* = −15→15
Absorption correction: multi-scan (*SCALEPACK*; Otwinowski & Minor, 1997)	*k* = −11→11
*T*_min_ = 0.966, *T*_max_ = 0.979	*l* = −25→25
17805 measured reflections	

### Refinement

**Table:**

Refinement on *F*^2^	Secondary atom site location: difference Fourier map
Least-squares matrix: full	Hydrogen site location: inferred from neighbouring sites
*R*[*F*^2^ > 2σ(*F*^2^)] = 0.051	H-atom parameters constrained
*wR*(*F*^2^) = 0.123	*w* = 1/[σ^2^(*F*_o_^2^) + (0.046*P*)^2^ + 0.4973*P*] where *P* = (*F*_o_^2^ + 2*F*_c_^2^)/3
*S* = 1.02	(Δ/σ)_max_ < 0.001
4690 reflections	Δρ_max_ = 0.36 e Å^−^^3^
139 parameters	Δρ_min_ = −0.32 e Å^−^^3^
0 restraints	Absolute structure: Flack (1983), 1953 Bijvoet pairs
0 constraints	Flack parameter: 0.35 (15)
Primary atom site location: structure-invariant direct methods	

### Special details

**Table:**

Geometry. All e.s.d.'s (except the e.s.d. in the dihedral angle between two l.s. planes) are estimated using the full covariance matrix. The cell e.s.d.'s are taken into account individually in the estimation of e.s.d.'s in distances, angles and torsion angles; correlations between e.s.d.'s in cell parameters are only used when they are defined by crystal symmetry. An approximate (isotropic) treatment of cell e.s.d.'s is used for estimating e.s.d.'s involving l.s. planes.

### Fractional atomic coordinates and isotropic or equivalent isotropic displacement parameters (Å^2^)

**Table:**

	*x*	*y*	*z*	*U*_iso_*/*U*_eq_	
C1	0.67962 (16)	0.2773 (3)	0.66604 (9)	0.0270 (3)	
C2	0.77829 (16)	0.2789 (3)	0.70632 (9)	0.0252 (3)	
C3	0.89002 (14)	0.2769 (3)	0.75911 (9)	0.0218 (3)	
C4	1.01780 (16)	0.2851 (3)	0.72849 (9)	0.0262 (3)	
H4	1.0316	0.2956	0.6719	0.031*	
C5	1.12430 (15)	0.2779 (3)	0.78050 (10)	0.0265 (3)	
H5	1.2111	0.2842	0.7602	0.032*	
C6	1.10107 (14)	0.2614 (3)	0.86239 (9)	0.0234 (3)	
C7	0.97590 (14)	0.2541 (3)	0.89480 (9)	0.0242 (3)	
H7	0.9629	0.2433	0.9515	0.029*	
C8	0.87080 (14)	0.2629 (3)	0.84253 (9)	0.0225 (3)	
H8	0.7843	0.2594	0.8635	0.027*	
C9	0.5612 (2)	0.2384 (5)	0.50094 (10)	0.0415 (5)	
H9A	0.6112	0.1226	0.4926	0.062*	
H9B	0.6129	0.3459	0.4825	0.062*	
H9C	0.4794	0.2322	0.4702	0.062*	
C10	0.4263 (2)	0.0658 (3)	0.64911 (14)	0.0376 (5)	
H10A	0.4097	0.0836	0.707	0.056*	
H10B	0.475	−0.0512	0.6409	0.056*	
H10C	0.3429	0.0593	0.6201	0.056*	
C10B	0.4280 (2)	0.4829 (3)	0.62787 (15)	0.0394 (5)	
H10D	0.477	0.5912	0.6075	0.059*	
H10E	0.4124	0.4982	0.686	0.059*	
H10F	0.3439	0.4742	0.5996	0.059*	
N1	1.21298 (13)	0.2499 (3)	0.91772 (9)	0.0345 (3)	
O1	1.32160 (13)	0.2830 (3)	0.89090 (11)	0.0531 (4)	
O2	1.19270 (16)	0.2043 (4)	0.98780 (9)	0.0670 (7)	
Si1	0.52365 (4)	0.26554 (7)	0.61012 (3)	0.02349 (11)	

### Atomic displacement parameters (Å^2^)

**Table:**

	*U*^11^	*U*^22^	*U*^33^	*U*^12^	*U*^13^	*U*^23^
C1	0.0248 (7)	0.0341 (10)	0.0221 (7)	0.0007 (8)	−0.0007 (6)	−0.0014 (7)
C2	0.0248 (7)	0.0291 (9)	0.0219 (6)	0.0008 (7)	−0.0009 (5)	−0.0006 (7)
C3	0.0208 (6)	0.0240 (8)	0.0207 (6)	0.0008 (7)	−0.0020 (5)	0.0005 (6)
C4	0.0247 (7)	0.0345 (9)	0.0194 (6)	0.0007 (8)	0.0022 (6)	0.0026 (6)
C5	0.0189 (6)	0.0334 (10)	0.0270 (7)	−0.0001 (7)	0.0027 (5)	0.0008 (8)
C6	0.0183 (6)	0.0274 (9)	0.0245 (6)	0.0001 (8)	−0.0048 (5)	−0.0022 (8)
C7	0.0223 (6)	0.0311 (8)	0.0192 (5)	0.0013 (8)	−0.0001 (5)	−0.0013 (7)
C8	0.0177 (6)	0.0285 (9)	0.0212 (6)	−0.0005 (7)	0.0001 (5)	−0.0016 (7)
C9	0.0392 (9)	0.0626 (15)	0.0228 (7)	−0.0031 (12)	−0.0018 (7)	−0.0023 (10)
C10	0.0296 (10)	0.0364 (12)	0.0467 (12)	0.0002 (8)	0.0011 (9)	0.0090 (9)
C10B	0.0310 (10)	0.0343 (11)	0.0530 (13)	0.0074 (8)	−0.0112 (10)	−0.0091 (9)
N1	0.0235 (6)	0.0442 (10)	0.0357 (7)	0.0034 (8)	−0.0094 (6)	−0.0075 (9)
O1	0.0217 (6)	0.0733 (12)	0.0641 (10)	−0.0078 (7)	−0.0111 (7)	0.0065 (11)
O2	0.0388 (8)	0.136 (2)	0.0264 (7)	0.0166 (12)	−0.0122 (6)	−0.0023 (10)
Si1	0.02129 (18)	0.0293 (2)	0.01984 (18)	0.00030 (19)	−0.00383 (15)	−0.00043 (19)

### Geometric parameters (Å, º)

**Table:**

C1—C2	1.209 (2)	C9—Si1	1.8560 (18)
C1—Si1	1.8450 (17)	C9—H9A	0.98
C2—C3	1.438 (2)	C9—H9B	0.98
C3—C8	1.397 (2)	C9—H9C	0.98
C3—C4	1.402 (2)	C10—Si1	1.853 (2)
C4—C5	1.388 (2)	C10—H10A	0.98
C4—H4	0.95	C10—H10B	0.98
C5—C6	1.380 (2)	C10—H10C	0.98
C5—H5	0.95	C10B—Si1	1.856 (2)
C6—C7	1.388 (2)	C10B—H10D	0.98
C6—N1	1.4671 (19)	C10B—H10E	0.98
C7—C8	1.380 (2)	C10B—H10F	0.98
C7—H7	0.95	N1—O1	1.219 (2)
C8—H8	0.95	N1—O2	1.221 (2)
			
C2—C1—Si1	176.09 (16)	H9A—C9—H9C	109.5
C1—C2—C3	175.90 (18)	H9B—C9—H9C	109.5
C8—C3—C4	119.38 (13)	Si1—C10—H10A	109.5
C8—C3—C2	119.25 (14)	Si1—C10—H10B	109.5
C4—C3—C2	121.36 (14)	H10A—C10—H10B	109.5
C5—C4—C3	120.35 (14)	Si1—C10—H10C	109.5
C5—C4—H4	119.8	H10A—C10—H10C	109.5
C3—C4—H4	119.8	H10B—C10—H10C	109.5
C6—C5—C4	118.44 (14)	Si1—C10B—H10D	109.5
C6—C5—H5	120.8	Si1—C10B—H10E	109.5
C4—C5—H5	120.8	H10D—C10B—H10E	109.5
C5—C6—C7	122.73 (14)	Si1—C10B—H10F	109.5
C5—C6—N1	118.86 (13)	H10D—C10B—H10F	109.5
C7—C6—N1	118.42 (14)	H10E—C10B—H10F	109.5
C8—C7—C6	118.29 (13)	O1—N1—O2	123.47 (16)
C8—C7—H7	120.9	O1—N1—C6	118.21 (16)
C6—C7—H7	120.9	O2—N1—C6	118.31 (15)
C7—C8—C3	120.81 (13)	C1—Si1—C10	108.92 (10)
C7—C8—H8	119.6	C1—Si1—C10B	109.77 (10)
C3—C8—H8	119.6	C10—Si1—C10B	107.68 (10)
Si1—C9—H9A	109.5	C1—Si1—C9	108.27 (8)
Si1—C9—H9B	109.5	C10—Si1—C9	111.68 (12)
H9A—C9—H9B	109.5	C10B—Si1—C9	110.51 (12)
Si1—C9—H9C	109.5		
			
C8—C3—C4—C5	0.5 (3)	C6—C7—C8—C3	0.6 (3)
C2—C3—C4—C5	−178.28 (19)	C4—C3—C8—C7	−1.0 (3)
C3—C4—C5—C6	0.4 (3)	C2—C3—C8—C7	177.76 (18)
C4—C5—C6—C7	−0.9 (3)	C5—C6—N1—O1	10.6 (3)
C4—C5—C6—N1	178.80 (18)	C7—C6—N1—O1	−169.7 (2)
C5—C6—C7—C8	0.3 (3)	C5—C6—N1—O2	−168.0 (2)
N1—C6—C7—C8	−179.33 (18)	C7—C6—N1—O2	11.7 (3)
